# Association between malnutrition and leucopenia in patients with osteosarcoma

**DOI:** 10.3389/fnut.2022.899501

**Published:** 2022-07-28

**Authors:** Haixiao Wu, Shu Li, Yile Lin, Jun Wang, Vladimir P. Chekhonin, Karl Peltzer, Vladimir P. Baklaushev, Kirellos Said Abbas, Jin Zhang, Huiyang Li, Chao Zhang

**Affiliations:** ^1^Tianjin Medical University Cancer Institute and Hospital, National Clinical Research Center for Cancer, Key Laboratory of Cancer Prevention and Therapy, Tianjin’s Clinical Research Center for Cancer, Tianjin, China; ^2^The Sino-Russian Joint Research Center for Bone Metastasis in Malignant Tumor, Tianjin, China; ^3^School of Management, Tianjin University of Traditional Chinese Medicine, Tianjin, China; ^4^Department of Orthopaedic Surgery, Tianjin Medical University General Hospital, Tianjin, China; ^5^Department of Oncology, Radiology and Nuclear Medicine, Medical Institute of Peoples’ Friendship University of Russia, Moscow, Russia; ^6^Department of Basic and Applied Neurobiology, Federal Medical Research Center for Psychiatry and Narcology, Moscow, Russia; ^7^Department of Psychology, University of the Free State, Bloemfontein, South Africa; ^8^Federal Research and Clinical Center of Specialized Medical Care and Medical Technologies, Federal Biomedical Agency of the Russian Federation, Moscow, Russia; ^9^Faculty of Medicine, Alexandria University, Alexandria, Egypt; ^10^Department of Obstetrics and Gynecology, Tianjin Medical University General Hospital, Tianjin, China

**Keywords:** leucopenia, malnutrition, osteosarcoma, chemotherapy, retrospective study

## Abstract

**Background and aim:**

Leucopenia (LP) greatly limits the efficacy of chemotherapy in osteosarcoma patients. This study aimed to evaluate the nutritional status of osteosarcoma patients before chemotherapy, assess the risk of LP during the perichemotherapy period, and explore the association between malnutrition and LP.

**Materials and methods:**

This study retrospectively analyzed osteosarcoma patients treated in the Tianjin Medical University Cancer Institute and Hospital, China, between January 2009 and December 2020 according to the inclusion and exclusion criteria. Malnutrition in adolescents (5 to 19 years old) and adults (≥20 years old) was diagnosed using WHO AnthroPlus software (version 1.0.4) and Global Leadership initiative on Malnutrition (GLIM), respectively. According to the diagnostic criteria of LP in CTCAE 5.0, patients were divided into the LP group and the non-LP group.

**Results:**

A total of 245 osteosarcoma patients were included. The incidence of malnutrition was 49.0%, and the incidence of LP was 51.8%. The incidence of malnutrition in adolescent patients was 53.1%, and their incidence of LP was 55.2%; the incidence of malnutrition in adult patients was 43.1%, and their incidence of LP was 47.1%. Logistic regression analysis showed that malnutrition before chemotherapy was an independent risk factor for the occurrence of LP after chemotherapy (OR = 6.85, 95% CI = 2.16-25.43; and OR = 35.03, 95% CI = 6.98-238.46 in mildly and severely malnourished young patients; OR = 6.06; 95% CI = 1.43-30.16; and OR = 38.09, 95% CI = 7.23-285.78 in mildly and severely malnourished adult patients, respectively). The results showed that age and nutritional status had a joint effect on the occurrence of LP.

**Conclusion:**

The nutrition status of osteosarcoma patients before chemotherapy is significantly correlated with the occurrence and severity of LP during peri-chemotherapy period. During osteosarcoma chemotherapy, necessary nutritional support should be given to patients of different ages to correct their malnutrition status in a timely manner, ultimately improving the efficacy of chemotherapy and the prognosis of patients.

## Introduction

Osteosarcoma is the most common primary malignant bone tumor and is common in adolescents, with an incidence of approximately 3-4 cases per million people (1). The clinical manifestations of osteosarcoma are mostly pain in the affected limbs, local mass, limb dysfunction, and pathological fracture. The treatment of osteosarcoma is based on clinical and pathological staging, combined with neoadjuvant chemotherapy, surgery, and adjuvant chemotherapy. The treatment effect of patients has been significantly improved, and the 5-year survival rate of osteosarcoma patients without distant metastasis has reached 70% (2, 3).

Chemotherapeutic drugs for osteosarcoma are highly toxic and can cause severe visceral, bone marrow, and nerve-related complications. Leucopenia (LP) is one of the most common complications of osteosarcoma during the perichemotherapy period. Previous research reports showed that during high-dose methotrexate (MTX) treatment, 16.8% of tumor patients developed LP (4). The incidence of grade II LP during cisplatin treatment is 26%, and that of grade III LP is 5% (5). Severe LP can significantly increase the risk of secondary infection, delay the progression of sequential chemotherapy, and increase the difficulty of treating osteosarcoma. Therefore, the prevention and treatment of LP can effectively ensure the implementation of osteosarcoma chemotherapy, thereby improving the treatment effect of osteosarcoma.

Malnutrition and attenuation of organ function occur in tumor patients due to their reduced energy intake, negative nitrogen balance, and increased resting energy consumption (6). The clinical manifestations include reduced body weight, decreased muscle mass, and reduced immunity, which significantly reduce the tolerance to treatment and the quality of life of patients (7–9). Malnutrition at the initial diagnosis is one of the main factors affecting the prognosis of patients with malignant tumors (10), and it is significantly associated with length of hospital stay, surgical complications (11, 12), toxic reactions to chemotherapy drugs (13), tumor recurrence (14), and early death (15). In one study, 48.28% of patients with bone and soft-tissue sarcoma were diagnosed with malnutrition at the time of initial diagnosis (16). In adolescent patients with solid tumors, the incidence of malnutrition at initial diagnosis can be as high as 50%. With the advancement of clinical treatment, the proportion of malnourished patients shows an increasing trend (17, 18).

The diagnostic criteria for malnutrition differ between adolescents and adults. The World Health Organization (WHO) has developed the WHO AnthroPlus software (version 1.0.4) as a common clinical diagnostic tool for malnutrition in adolescents (5-19 years) by analyzing the average nutritional status of adolescents of different ethnic groups (19). In 2016, the Global Clinical Nutrition Association launched a diagnostic criterion for malnutrition in adults, Global Leadership initiative on Malnutrition (GLIM), and it recommended that this consensus be used as the basis for the diagnosis of malnutrition in adults with sarcopenia, cachexia, and frailty (9).

In summary, in view of the onset characteristics of osteosarcoma malnutrition and the high risk of LP during the perichemotherapy period, this retrospective analysis aimed to evaluate the nutritional status of osteosarcoma patients before chemotherapy and the risk of LP during chemotherapy. At the same time, the association between nutritional status and the occurrence of LP in osteosarcoma populations of different ages during the perichemotherapy period was analyzed to explore the importance of malnutrition risk screening in osteosarcoma patients, thereby to achieve stratified management and target treatment for osteosarcoma patients undergoing chemotherapy.

## Materials and methods

### Inclusion and exclusion criteria

**FIGURE 1:**
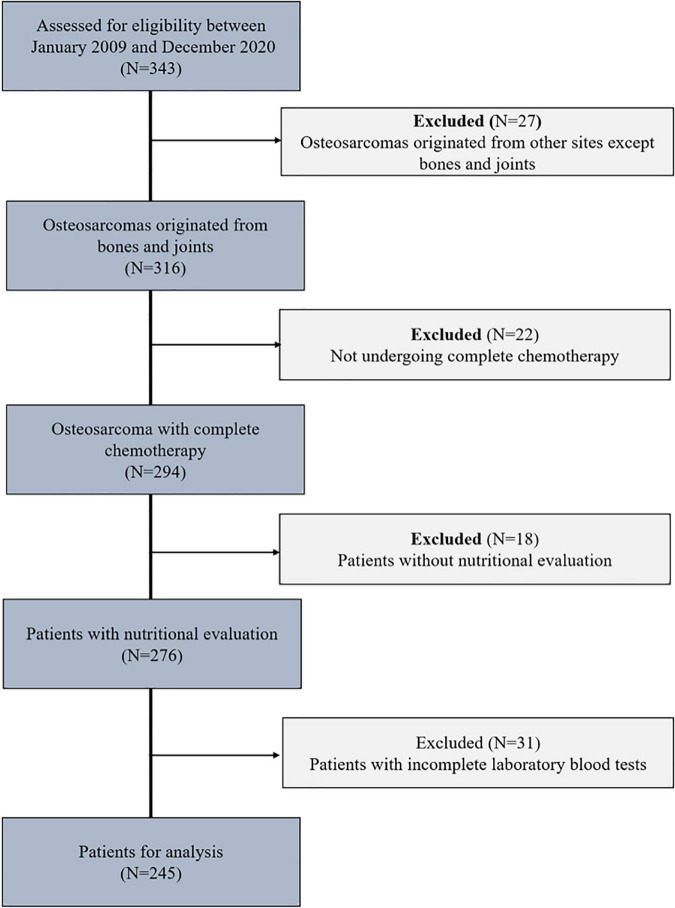
Flowchart of the enrolled patients.

We retrospectively analyzed 343 osteosarcoma patients who received comprehensive treatment at the Tianjin Medical University Cancer Institute and Hospital, China, between January 2009 and December 2020. Inclusion criteria: Patients who were diagnosed with primary osteosarcoma by pathology, had a performance status of 0 to 4 points in the WHO diagnosis of tumor patients, had no significant abnormalities in routine blood tests or liver or kidney function before each chemotherapy session, and had complete clinical information. Exclusion criteria: patients with unclear diagnosis by pathology and/or multiple malignancies, patients with congenital malnutrition and/or developmental delay, patients with osteosarcoma, paraosseous osteosarcoma, or secondary osteosarcoma who did not receive complete chemotherapy, patients with bone marrow dysfunction suggested by prechemotherapy blood routine results, and patients with missing clinical data (Figure 1).

According to the clinical and pathological characteristics of the patients, the following characteristics were included: age, body mass index (BMI), nutritional status, performance status, lesion location, pathological subtype, surgical staging by the Musculoskeletal Tumor Society, and Ki-67. Peripheral venous blood samples were collected from the patients after admission. Laboratory methods were used to determine the complete blood count and basic biochemical indicators as well as the white blood cell (WBC) count within 1 week after chemotherapy. BMI was calculated as: BMI = weight/height^2^ (weight unit: kilogram; height unit: meter).

In this study, the Eastern Cooperative Oncology Group (ECOG) scoring system was used to evaluate the PS (performance status, PS) of osteosarcoma patients before chemotherapy (20): 0 points, normal activities; 1 point, uncomfortable symptoms, no need to stay in bed, able to take care of themselves; 2 points, wake-up and exercise time >50%, occasionally need help; 3 points, wake-up time <50%, need care; 4 points, confined to bed.

### Chemotherapy regimen for osteosarcoma patients

After the diagnosis of osteosarcoma, the patients were given the following neoadjuvant chemotherapy regimen: MAP regimen: methotrexate 8–12 g/m^2^ on days 1 and 8, cisplatin 90 mg/m^2^ on day 15, doxorubicin 75 mg/m^2^ on day 15. After a 28-day break, this regimen was repeated once for a total of two times.

After the completion of neoadjuvant chemotherapy, patients were given limb salvage or amputation, and the adjuvant chemotherapy regimen was chosen by analyzing the tumor necrosis rate. The same treatment as the neoadjuvant chemotherapy regimen was given when the tumor necrosis rate was >90%; the chemotherapy regimen was changed when the tumor necrosis rate was <90%: MAPI regimen: ifosfamide 2 g/m^2^ on day 1-5; methotrexate 8–12 g/m^2^ on day 8; cisplatin 90 mg/m^2^ on day 21, doxorubicin 75 mg/m^2^ on day 21. After a 28-day break, the regimen was repeated. A total of four cycles of adjuvant chemotherapy were performed.

Standard hydration, alkalization, blood concentration monitoring, urine pH measurement, and leucovorin detoxification were done during the treatment with methotrexate. Standard hydration, alkalization, and cardioprotective agent were given during the treatment with cisplatin and doxorubicin. Mesna was given as adjuvant therapy during the treatment with ifosfamide. In addition, the patients were given stomach protection, antiemetics, vitamin B6, diuretics, and nutritional support treatment during chemotherapy. The total fluid of the patient during each chemotherapy was approximately 3,000-3,500 ml.

If the patient had fewer than 3.0 × 10^9^/L WBCs or fewer than 75 × 10^9^/L platelets within 1 week after chemotherapy or had other severe liver or kidney toxicities, we recommended delaying the subsequent course of treatment.

## Evaluation of the nutritional status of osteosarcoma patients

The Anthro software of the World Health Organization (version 1.0.4; Department of Nutrition, WHO) was used to assess the nutritional status for children and adolescents (5 to 19 years old), weight-for-age (W/A) was calculated for patients aged 5 to 9 years, and BMI-for-age was the recommended indicator for patients aged 10-19 years. The values obtained were expressed as *z*-scores, and the nutritional status was classified according to the WHO recommendation for each age group. Malnutrition was considered as values *Z*-score < −2SD, either for W/A or BMI/A in all age groups. The severity of malnutrition was classified as follows (21):

• Above adequate (normal nutrition) - patients with a *Z*-score more than one SD above the mean BMI for age, (*Z*-score >1SD);
• Adequate (normal nutrition) - patients with a *Z*-score between two SD below and one SD above the mean BMI for age, (−2SD < *Z*-score < 1SD);
• Mild malnutrition - patients with a *Z*-score more than two standard deviations (SD) below the mean BMI for age, (*Z*-score < −2SD);
• Severe malnutrition - patients with a *Z*-score more than three standard deviations (SD) below the mean BMI for age, (*Z*-score < −3SD).

Malnutrition was diagnosed by the GLIM criteria in adult patients ≥20 years of age. The specific diagnostic criteria were evaluated using the phenotypic and etiologic criteria of GLIM (9).

• **Phenotypic:**

(1) Unintentional weight loss (>5% in 6 months);

(2) Low BMI (<20 kg/m^2^ if <70 years and <22 kg/m^2^ if >70 years);

(3) Reduction of muscle mass.

• **Etiologic:**

Reduced intake (> 50% of energy intake during the last pre-admission week) or inflammatory response of the disease (C-reactive protein (CRP) values >5 mg/dL).

According to the GLIM diagnostic criteria, the diagnosis of malnutrition can be made when the patient meets at least one phenotypic and one etiologic criterion. Subsequently, the severity of malnutrition was classified according to phenotype: (1). Mild malnutrition: the body weight of the patient had decreased by 5-10% in the past 6 months or the BMI was 18.5-20 m/kg^2^ (age <70 years) or <22 m/kg^2^ (>70 years old). (2). Severe malnutrition: the weight of the patient had decreased >10% in the past 6 months, or BMI <18.5 m/kg^2^ (<70 years old) or BMI < 20 m/kg^2^ (> 70 years old).

### Diagnostic criteria for leucopenia in osteosarcoma patients

Chemotherapy can cause nausea, vomiting, myelosuppression, hepatorenal toxicity, cardiotoxicity, and nervous system toxicity. In clinical practice, CTCAE (Common Terminology Criteria for Adverse Events, CTCAE) was often used to judge the level of adverse events in patients to facilitate symptomatic treatment. According to the diagnostic criteria for LP in CTCAE 5.0, LP was defined as the WBC count <3.0 × 10^9^/L within 1 week after chemotherapy. Its severity was classified as follows (22, 23): (1). No leucopenia (grade 0): WBC ≥3.0 × 10^9^/L. (2). Mild leucopenia (grade 1-2): 2.0 × 10^9^/L ≤ WBC < 3.0 × 10^9^/L. (3). Severe leucopenia (grade 3-4): WBC < 2.0 × 10^9^/L.

### Statistical analysis

Quantitative data were statistically described as median (interquartile range [IQR]), and the Kruskal–Wallis test was used for intergroup comparisons, including age and serological indicators. Qualitative data are expressed as the number of cases (n) or rate (%), and the chi-squared test was used for intergroup comparisons. The patients were divided into the LP group and the non-LP group. Spearman rank correlation analysis was used to calculate the correlation coefficients between different specific indicators of malnutrition (adults: BMI and CRP; adolescents: *Z*-score) and WBC count after chemotherapy. A receiver operating characteristic (ROC) curve was plotted to determine the cut-off values of different specific indicators of malnutrition (adults: BMI and CRP; adolescents: *Z*-score) for predicting the occurrence of LP and to evaluate their predictive efficacy. Logistic regression analysis was used to determine the odds ratio (OR) and 95% confidence interval (CI) of malnutrition and the risk of LP. R 3.6.3 software was used for data processing and analysis, and *p* < 0.05 was considered statistically significant.

## Results

A total of 343 osteosarcoma patients were treated at the Tianjin Medical University Cancer Institute and Hospital from 2009 to 2020. Twenty-seven patients were excluded due to extraskeletal osteosarcoma, 22 patients were excluded for not undergoing complete chemotherapy, 18 patients were excluded due to a lack of nutritional assessment data, and 31 patients were excluded due to a lack of laboratory blood test data. In summary, after applying the inclusion and exclusion criteria, a total of 245 osteosarcoma patients were included in this study (Figure 1).

### Description of the characteristics of the included patients

The analysis of the baseline information on the demographic, clinical, pathological, and laboratory data of the included patients showed that the average age of the patients was 23.46 ± 14.98 years old. There were 144 males and 101 females. There were 143 adolescent patients and 102 adult patients. Osteosarcoma lesions were located in the distal femur in 188 cases, in the proximal tibia in 28 cases, and in the proximal humerus, ilium, clavicle, and fibula in 29 cases. The pathological types of osteosarcoma included 164 cases of osteogenic type, 44 cases of chondrogenic type, 12 cases of fibrous type, and 25 cases of other pathological subtypes (telangiectatic type, small round cell type, and malignant transformation of giant cell tumors of bone). According to the Musculoskeletal Tumor Society surgical staging, there were 24 cases of grade IIA, 192 cases of grade IIB, and 29 cases of grade III. Before the first chemotherapy, 125 patients had normal nutritional status, and 120 patients had malnutrition; 117 patients had normal performance status, and 128 patients had abnormal performance status.

The incidence of malnutrition in all included osteosarcoma patients was 49.0%, and the incidence of LP was 51.8%. Malnutrition occurred in 53.1% of adolescent patients, and the incidence of LP was 55.2%; 43.1% of adult patients were diagnosed with malnutrition, and the incidence of LP was 47.1% (Table 1). In addition, the analysis of the baseline data of all osteosarcoma patients showed that osteosarcoma patients with lower BMI were more likely to suffer from LP after chemotherapy. When the serum protein level was lower, the probability of LP was higher (*p* = 0.003); the higher the performance status value and C-reactive protein value in adult patients, the higher the probability of LP (*p* = 0.049 and *p* < 0.001, respectively).

**TABLE 1:** Comparison between the leucopenia profiles on socio-demographics, clinical, pathologic and nutrient in different population.

Total population	Overall (*N* = 245)	Adolescent			Adult		
			
		Non-LP (*n* = 64)	LP (*n* = 79)	*p*-value	Non-LP (*n* = 54)	LP (*n* = 48)	*P*-value
Age (years)	18.00 [14.00, 28.00]	13.50 [12.00, 16.00]	14.00 [11.00, 16.00]	0.545	33.50 [25.00, 48.00]	30.50 [24.75, 44.75]	0.462
Sex, female, n (%)	101 (41.2)	24 (37.5)	38 (48.1)	0.27	19 (35.2)	20 (41.7)	0.64
BMI (kg/m^2^)	17.79 [15.00, 20.43]	17.08 [14.83, 19.75]	14.79 [14.05, 16.23]	<0.001	20.57 [20.31, 20.99]	18.56 [17.61, 20.31]	<0.001
Nutrient, n (%)				<0.001			<0.001
Normal	125 (51.0)	44 (68.8)	23 (29.1)		43 (79.6)	15 (31.2)	
Mild	65 (26.5)	17 (26.6)	29 (36.7)		8 (14.8)	11 (22.9)	
Severe	55 (22.4)	3 (4.7)	27 (34.2)		3 (5.6)	22 (45.8)	
PS, n (%)				0.073			0.049
0 score	117 (47.8)	35 (54.7)	27 (34.2)		36 (66.7)	19 (39.6)	
1 score	40 (16.3)	10 (15.6)	14 (17.7)		7 (13.0)	9 (18.8)	
2 score	71 (29.0)	16 (25.0)	29 (36.7)		9 (16.7)	17 (35.4)	
3 score	17 (6.9)	3 (4.7)	9 (11.4)		2 (3.7)	3 (6.2)	
Location, n (%)				0.592			0.845
Distal femur	186 (75.9)	43 (67.2)	58 (73.4)		46 (85.2)	39 (81.2)	
Proximal tibia	30 (12.2)	12 (18.8)	10 (12.7)		4 (7.4)	4 (8.3)	
Other	29 (11.8)	9 (14.1)	11 (13.9)		4 (7.4)	5 (10.4)	
Pathology, n (%)				0.861			0.217
Osteogenic	164 (66.9)	44 (68.8)	51 (64.6)		38 (70.4)	31 (64.6)	
Chondrogenic	44 (18.0)	9 (14.1)	12 (15.2)		9 (16.7)	14 (29.2)	
Other	37 (15.1)	11 (17.2)	16 (20.3)		7 (13.0)	3 (6.2)	
MSTS staging, n (%)				0.345			0.903
IIA	24 (9.8)	8 (12.5)	7 (8.9)		5 (9.3)	4 (8.3)	
IIB	192 (78.4)	51 (79.7)	60 (75.9)		42 (77.8)	39 (81.2)	
III	29(11.8)	5 (7.8)	12 (15.2)		7 (13.0)	5 (10.4)	
CRP (mg/L)	7.50 [2.63, 12.00]	8.19 [2.48, 12.38]	7.55 [3.02, 12.30]	0.955	3.20 [2.32, 6.15]	10.25 [7.00, 15.33]	<0.001
WBC before (× 10^9^/L)	6.85 [5.76, 8.20]	6.99 [5.80, 8.51]	6.86 [5.88, 8.19]	0.713	6.85 [5.72, 8.08]	6.70 [5.72, 8.56]	0.96
Alb (g/L)	28.90 [27.90, 29.90]	29.60 [28.28, 39.40]	28.50 [28.05, 29.70]	0.013	29.30 [28.13, 34.68]	28.40 [27.00, 29.35]	0.008

### Relationships of malnutrition to white blood cell count after chemotherapy, and the occurrence of leucopenia

**FIGURE 2:**
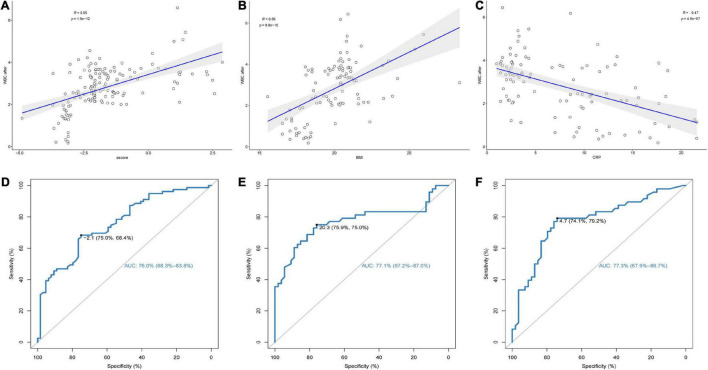
(1) The correlation between **(A)** *Z*-score of adolescents, **(B)** body mass index (BMI) of adults, **(C)** C-reactive protein (CRP) of adults, and white blood cell (WBC) levels after chemotherapy. (2) Receiver-operating characteristics (ROC) curves identifying discrimination thresholds of **(D)** *Z*-score of adolescents, **(E)** body mass index (BMI) of adults, **(F)** C-reactive protein (CRP) of adults for Leucopenia (LP).

In adolescent patients, there was a positive correlation between WBC count and *Z* score after chemotherapy (*R* = 0.55, *p* < 0.001) (Figure 2A). In adult patients, the WBC count was positively correlated with BMI level after chemotherapy (*R* = 0.56, *p* < 0.001) and negatively correlated with CRP level (*R* = −0.47, *p* < 0.001) (Figures 2B,C). The ROC curve showed that different specific indicators of malnutrition had good predictive power for the occurrence of LP. The area under the curve (AUC) for predicting the occurrence of LP in adolescent patients using the *Z*-score was 76.0% (with a cut-off value of −2.1) (Figure 2D), the AUC for predicting the occurrence of LP in adult patients using BMI was 77.1% (with a cut-off value of 20.3) (Figure 2E), and the AUC for predicting the occurrence of LP in adult patients using CRP was 77.3% (with a cut-off value of 4.7) (Figure 2F). These findings are consistent with the criteria used to determine malnutrition in the current literature.

### Association analysis of nutritional status and leucopenia

The LP multivariate-adjusted OR (95% CI) of adolescent patients with mild/severe malnutrition vs. non-malnutrition showed that the presence of malnutrition before chemotherapy was an independent risk factor for the development of LP after chemotherapy (OR = 6.85, 95% CI = 2.16-25.43; and OR = 35.03, 95% CI = 6.98-238.46 in mildly and severely malnourished young patients, respectively). The multivariate adjusted OR (95% CI) of LP in adult patients with mild/severe malnutrition and non-malnutrition showed that the presence of malnutrition before chemotherapy was independently associated with an increased risk of LP (OR = 6.06, 95% CI = 1.43-30.16; and OR = 38.09, 95% CI = 7.23-285.78 in mildly and severely malnourished adult patients, respectively). In summary, the nutritional status of adolescents and adults with osteosarcoma before chemotherapy was highly correlated with the occurrence of LP and was an independent risk factor (Table 2).

**TABLE 2:** The associations between the nutritional status and the risk of LP in patients with osteosarcoma during chemotherapy (*n* = 245).

Characteristic	Patients at risk, (N)	Events, (*n*)	Model A*		Model B^†^		Model C^‡^	
							
			Univariate OR (95% CI)	*P*-value	Multivariable OR (95% CI)	*P*-value	Multivariable OR (95% CI))	*P*-value
**Adolescent**								
Nutrient status Normal	67	23	1 (Reference)		1 (Reference)		1 (Reference)	
Mild	46	29	3.26 (1.51-7.27)	0.003	3.39 (1.52-7.81)	0.003	6.85 (2.16-25.43)	0.002
Severe	30	27	17.22 (5.37-77.63)	<0.001	17.33 (5.31-79.27)	<0.001	35.03 (6.98-238.46)	<0.001
**Adult**								
Nutrient status Normal	58	15	1 (Reference)		1 (Reference)		1 (Reference)	
Mild	19	11	3.94 (1.35-12.06)	0.013	5.24 (1.62-18.56)	0.007	6.06 (1.43-30.16)	0.019
Severe	25	22	21.02 (6.21-98.27)	<0.001	26.55 (7.30-136.06)	<0.001	38.09 (7.23-285.78)	<0.001

*Model A: crude analysis; ^†^Model B: adjusted for sex and age; ^‡^Model C: adjusted for sex, age, BMI, PS, location, pathology, MSTS staging, Ki67, CRP, HGB, PLT, WBC before, and ALB.

### Association analysis of nutritional status and LP severity and frequency

Nutritional status of osteosarcoma patients was significantly association with both severity and frequency of LP occurrence after chemotherapy. Among adolescent osteosarcoma patients, the probability of non-LP was higher in patients with normal nutritional status, while the risk of severe LP was significantly increased in patients with severe malnutrition. The worse the nutritional status was, the increased number of LP in adolescent patients was found.

**FIGURE 3:**
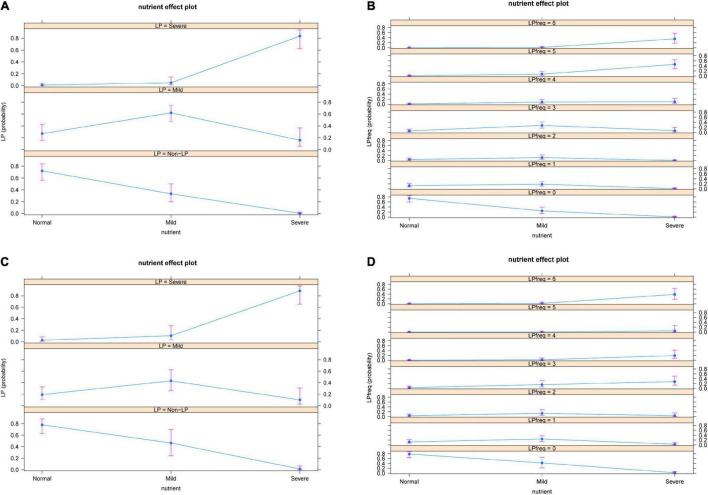
Severity **(A)** and frequency **(B)** of nutritional status and occurrence of LP in adolescent patients. Severity **(C)** and frequency **(D)** of nutritional status and occurrence of LP in adult patients. The results showed that the severity and frequency of LP increased with the deterioration of patients’ nutritional status. LP: Leucopenia.

Similar trend was observed in adult patients with osteosarcoma. The severity of LP increased with the deterioration of nutritional status. The probability of LP occurrence decreased in patients with normal nutritional status, while the incidence of LP was significantly increased in patients with severe malnutrition (Figure 3).

### Joint effect of the nutritional status of osteosarcoma patients

**FIGURE 4:**
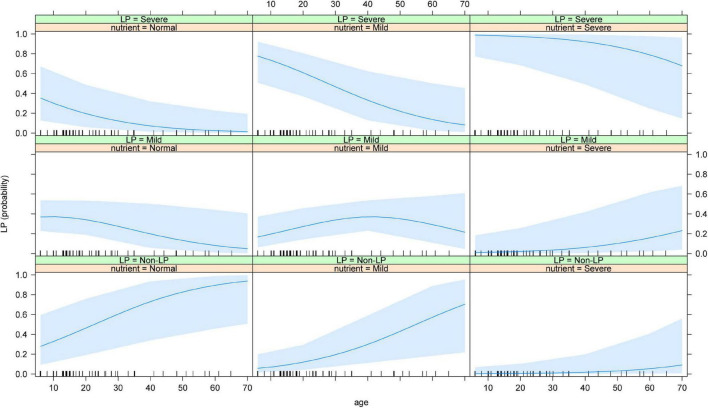
Joint effect of age and the nutritional status on the risk of LP. The results showed that the risk of severe LP increased with the increased age and the worse nutritional status. LP: Leucopenia.

We next conducted an in-depth analysis of the joint effects of age and nutritional status on the occurrence of LP. The results showed that in osteosarcoma patients with normal nutritional status, the probability of normal WBC count after chemotherapy was significantly higher than the probability of mild and severe LP with increasing age. In osteosarcoma patients with mild malnutrition, the probability of developing a normal WBC count after chemotherapy was significantly higher in adults than in adolescents, while the risk of developing severe LP decreased gradually with age, though the probability of developing mild LP did not differ significantly between adolescents and adults. In patients with severe malnutrition, the probability of developing normal WBC and mild malnutrition gradually increased with age, while the probability of developing severe LP gradually decreased (Figure 4).

## Discussion

Osteosarcoma is a highly malignant tumor of mesenchymal origin that lacks typical clinical manifestations, progresses rapidly, and is difficult to treat. The rapid growth of tumor tissues can also trigger the body to have a negative nitrogen balance, increased resting state metabolism, and systemic inflammatory responses, resulting in tumor-related malnutrition (9, 24, 25). In clinical practice, the doses of chemotherapeutic drugs for osteosarcoma are relatively high, the toxicity is relatively severe, and the risk of LP during the perichemotherapy period is relatively high.

This study used different malnutrition diagnostic systems to evaluate different populations with osteosarcoma. It was the first study in the world to analyze the relationship between malnutrition and the risk of LP after osteosarcoma chemotherapy. The incidence of malnutrition of the whole population at the time of initial diagnosis was 49.0%, and the incidence of LP was 51.8%. Among them, 53.1% of the patients in the adolescent group had malnutrition, and their incidence of LP was 55.2%; in the adult group, 43.1% of the patients had malnutrition, and their incidence of LP was 47.1%. The incidence of malnutrition in cancer patients during the initial diagnosis can be as high as 30-80%, and the risk of blood complications (LP and NP) after chemotherapy can reach 60.18% (10, 16, 26). After chemotherapy, severe malnutrition occurred in 25% of cancer patients, and mild to moderate malnutrition occurred in 42% of patients (27). A previous study further confirmed the prevalence of nutritional risk was 44% in cancer patients (28). Therefore, changes in body composition caused by malnutrition in cancer patients may be one of the important reasons for chemotherapy-related complications, which was consistent with this study.

At present, the exact mechanism of malnutrition and LP is not fully understood. With the rapid growth of tumors, various metabolic disorders can gradually appear in the body, resulting in sarcopenia (decreased skeletal muscle mass and strength), sarcopenic obesity, and osteoporosis (29, 30). In recent years, these factors have been considered important risk factors for the decline in physical strength during perichemotherapy period in cancer patients (31, 32). In addition, structural and cellular changes of the hematopoietic microenvironment in malnutrition contribute to bone marrow hypoplasia and arrests hematopoietic stem cells in G_0_/G_1_ cell cycle phases, which cause anemia, leukopenia, thrombocytopenia and impaired immune response (33, 34). Muscle and fat mass can be usually used for malnutrition evaluation. Reduced muscle mass and obesity were common in patients with malnutrition and cachexia. Patients with the lower muscle mass usually showed higher plasma concentrations of hydrophilic drugs, which resulted in the more severe toxicity. With the treatment of lipophilic drugs such as doxorubicin, the patients with low-fat mass presented with the increased toxicity due to the reduced volume of distribution (35). Thus, hematologic toxicity could be significantly increased.

This study found that LP was more likely to occur when the serum protein content was low. Malnourished cancer patients often develop hypoproteinaemia due to anorexia, inflammatory status, and metabolic changes, which significantly increase the risk of early death and secondary infection in cancer patients (36, 37).

Our study looking into the adolescent and adult patients with osteosarcoma throughout chemotherapy, for the first time, reported that both the severity and the frequency of LP were significantly correlated with nutritional status. The worse the nutritional status being found, the more risk of LP occurred and the more severe LP can be found in osteosarcoma patients. Previous studies have confirmed that, nutritional support therapy can significantly improve chemotherapy-related toxicity (hemato-, renal- and hepatotoxicity, etc.) (38). Thus, in order to reduce the risk of LP and improve the overall efficacy of chemotherapy, attention should be paid to the changes of nutritional status in patients with osteosarcoma during chemotherapy.

This study find that LP was rarer in osteosarcoma patients with normal nutritional status than in osteosarcoma patients with malnutrition, and it had no significant correlation with the age of the patients. Among patients with mild malnutrition, the proportion of LP in adolescent patients was significantly higher than that in adults, while there was no significant difference in the proportion of LP in the two groups of patients with severe malnutrition. Adolescent patients with bone or soft-tissue sarcoma have a higher risk of developing adverse blood reactions after chemotherapy. Especially for osteosarcoma patients, the treatment doses of methotrexate and cisplatin relatively higher at younger ages. The development of the hematopoietic system in young patients is often immature, which makes these patients more susceptible to the effects of chemotherapy drugs, resulting in adverse blood reactions. At the same time, the body composition of adolescent patients varies greatly. The contents of lean body tissue and adipose tissue affect the distribution and metabolism of chemotherapy drugs, which can significantly change the clearance rate of hydrophilic and/or lipophilic drugs from the body and inhibit bone marrow hematopoietic function (16, 18).

This study had certain limitations. First, this study was a single-center retrospective study, there might be a certain bias in sex and age. Future studies with larger sample size should be further performed to validate our conclusion. Second, we did not conduct an in-depth analysis of the different time points among LP episodes in patients during the peri-chemotherapy. Third, due to the limited sample size, we did not measure the reduction of muscle mass. The muscle mass can be a significant factor evaluating the nutrition status of patients. Finally, we found that nutrition therapy during chemotherapy can alleviate chemotherapy-induced complications such as decreased appetite, nausea, vomiting, and myelosuppression. Therefore, future research should be performed to explore the effect of nutrition therapy during osteosarcoma chemotherapy. The osteosarcoma patients may benefit from the reduced adverse events of nutrition.

## Conclusion

This study described the nutritional status of osteosarcoma patients in a single centre and evaluated the risk of malnutrition in osteosarcoma patients before treatment and LP during the perichemotherapy period. The association between nutritional status before chemotherapy and LP was explored. After adjusting for confounding variables, multivariate analysis showed that malnutrition was correlated with the risk of LP during perichemotherapy period and was an independent risk factor for LP. LP frequency and severity were proved to be correlated with the nutritional status of the osteosarcoma patients. The worse nutritional status can result in the increased risk of LP and the severe LP occurrence.

This study emphasized the importance of nutritional screening and evaluation for osteosarcoma patients perichemotherapy. For patients with malnutrition, early nutritional intervention should be encouraged to reduce the occurrence of LP, thereby ensuring their tolerance of chemotherapy.

## Data availability statement

The raw data supporting the conclusions of this article will be made available by the authors, without undue reservation.

## Ethics statement

The studies involving human participants were reviewed and approved by Ethical Review Board of Tianjin Cancer Institute and Hospital. Written informed consent for participation was not required for this study in accordance with the national legislation and the institutional requirements.

## Author contributions

CZ and HL proposed the idea and supervised the work. HW, SL, YL, and JW collected the data and analyzed it. VC, KP, and VB formulated the first draft. KA and JZ revised and edited the draft. All authors revised the manuscript critically.
